# Ion beam-induced shaping of Ni nanoparticles embedded in a silica matrix: from spherical to prolate shape

**DOI:** 10.1186/1556-276X-6-155

**Published:** 2011-02-18

**Authors:** Hardeep Kumar, Santanu Ghosh, Devesh Kumar Avasthi, Debdulal Kabiraj, Arndt Mücklich, Shengqiang Zhou, Heidemarie Schmidt, Jean-Paul Stoquert

**Affiliations:** 1 Nanostech Laboratory, Department of Physics, Indian Institute of Technology Delhi, New Delhi 110016, India; 2 Inter University Accelerator Centre, Aruna Asaf Ali Marg, New Delhi 110067, India; 3 Institute of Ion Beam Physics and Materials Research, Forschungszentrum Dresden-Rossendorf, P.O. Box 510119, 01314 Dresden, Germany; 4 Institut d'Electronique du Solide et des Systèmes, 23 rue du Loess, BP 20 CR, 67037 Strasbourg Cedex 2, France

## Abstract

Present work reports the elongation of spherical Ni nanoparticles (NPs) parallel to each other, due to bombardment with 120 MeV Au^+9 ^ions at a fluence of 5 × 10^13 ^ions/cm^2^. The Ni NPs embedded in silica matrix have been prepared by atom beam sputtering technique and subsequent annealing. The elongation of Ni NPs due to interaction with Au^+9 ^ions as investigated by cross-sectional transmission electron microscopy (TEM) shows a strong dependence on initial Ni particle size and is explained on the basis of thermal spike model. Irradiation induces a change from single crystalline nature of spherical particles to polycrystalline nature of elongated particles. Magnetization measurements indicate that changes in coercivity (*H*_c_) and remanence ratio (*M*_r_/*M*_s_) are stronger in the ion beam direction due to the preferential easy axis of elongated particles in the beam direction.

## Introduction

Metal nanoparticles (NPs) embedded in transparent matrices are the subject of large scientific and technological interest as they show significantly different properties as compared to their bulk counterpart [1,2]. The NP size and shape, orientation, interparticle separation and dielectric constant of the surrounding matrix are the crucial parameters which control their properties. Generally, the NP shape and orientation is difficult to control by synthesis parameters. One of the interesting aspects of shape anisotropy in noble metal NPs is the splitting of the surface plasmon resonance band [3-6], which can be tuned from visible to infrared region. Prolate-shaped NPs/nanorods show new and improved photonic, optoelectronic, and sensing properties as compared to spherical NPs [3,5]. On the other hand, an array of magnetic prolate-shaped NPs/nanorods with perpendicular magnetic anisotropy permits to overcome the problem of superparamagnetic instability arising due to the decrease in the particle size in magnetic recording media [6-8]. Another requirement for recording at high density with a minimum noise is to reduce the interaction between magnetic nanorods, which can be achieved by encapsulation of magnetic nanorods in a non-magnetic matrix. In literature, various methods are reported to prepare prolate-shaped NPs/nanorods, but the investigated methods yield randomly oriented structures (e.g., by chemical routes) [3], small areas (e.g., by electron or focused ion-beam lithography) [5,7] or are limited to a specific class of materials (e.g., porous alumina template growth) [6,8].

Swift heavy ion (SHI) irradiation is an important tool in the modification of materials and is extensively used to manipulate the matter at nanometer scale. One of the important effects of SHI irradiation is the anisotropic shape deformation of amorphous silica nanospheres to oblate shape [9,10] and crystalline metallic NPs, e.g., Co [11,12], Au [13-17], Ag [18-21], Pt [22,23], and FePt [24] embedded in silica matrix, to prolate shape. No shape deformation is observed for embedded Fe NPs in silica matrix by 120 MeV Au^9+ ^ions at a fluence of 3 × 10^13 ^ions/cm^2^, However, tilt of easy axis of magnetization [25,26] was observed and explained by ion hammering effect. The deformation behavior of silica nanospheres, i.e., expansion in the direction perpendicular to ion beam and shrinkage in the direction parallel to ion beam, is known under the name "hammering effect" and explained by the viscoelastic thermal spike model [27,28]. On the other hand, there is no consistent theory describing the shape deformation of metal NPs in amorphous silica matrix, but the suggested mechanisms include melting of NPs in thermal spike [29-31], creep deformation induced by an overpressure due to differences in volume expansion and compressibility of NP and silica matrix [11], and shear stress-driven deformation due to in-plane strain perpendicular to ion beam direction [14,16,22,23].

In the present work, we report the elongation/anisotropic shape deformation of Ni NPs from spherical to prolate ones under 120 MeV Au^+9 ^ion irradiation at fluence of 5 × 10^13 ^ions/cm^2^, where shape deformation strongly depends on the initial Ni particle size. Further, to understand the shape deformation process, simulations based on thermal spike model [29-31] were carried out and the effect of irradiation on structural and magnetic properties is presented.

### Experimental details

A set of thin films of silica containing Ni NPs (Ni-SiO_2 _nanogranular films) were synthesized by atom beam sputtering technique, as described elsewhere [32-35]. Silica and Ni were co-sputtered on thermally oxidized Si substrates mounted on a rotating sample holder. The relative area of silica and Ni chips exposed to the atom beam determines the concentration and size of Ni particles. In this study, the area of Ni was maintained to obtain ~10 at% Ni in the films. Ni-SiO_2 _nanogranular films were annealed in Ar-H_2 _(5%) atmosphere at 850°C (1 h) for promoting the growth of Ni particles and labeled as *pristine *film thereafter. The *pristine *film was irradiated at room temperature and at normal incidence with 120 MeV Au^+9 ^ions at a fluence of 5 × 10^13 ^ions/cm^2 ^in 15 UD Tandem Pelletron accelerator at the Inter University Accelerator Centre, New Delhi, India. The irradiation was performed in a high vacuum chamber with a base pressure of 2.8 × 10^-6 ^Torr. The beam current was kept <0.5 pnA (particle nano ampere) during irradiation to avoid heating of the film. The ion beam was uniformly scanned over 1 × 1 cm^2 ^area using an electromagnetic scanner. The range, electronic (*S*_e_) and nuclear (*S*_n_) stopping powers of 120 MeV Au^+9 ^ions in silica were calculated using SRIM 2006 code [36] and amount to ~15 μm, 14.7 keV/nm and 0.2 keV/nm, respectively. For such a large range, stopping powers can be considered constant over a film of few nanometers thickness. The composition and film thickness were measured by Rutherford backscattering spectrometry (RBS) using 1.7 MeV He^+ ^ions at a scattering angle of 170°. Magnetization curves were measured using a Quantum Design MPMS SQUID magnetometer with a maximum field of 2 T applied parallel (out-plane measurement) and perpendicular (in-plane measurement) to the ion beam direction. TEM measurements were used to evaluate the size and shape evolution of Ni NPs before and after irradiation. TEM samples were prepared in cross-sectional geometry using the conventional techniques and were analyzed in FEI Titan 80-300 microscope working at accelerating voltage of 300 kV.

## Results and discussion

The measured film thickness is ~150 nm with an average Ni atomic concentration of 10.5 ± 1% as estimated from fitting of RBS spectra using RUMP simulation code [37].

### Micro-structural study

**Figure 1:**
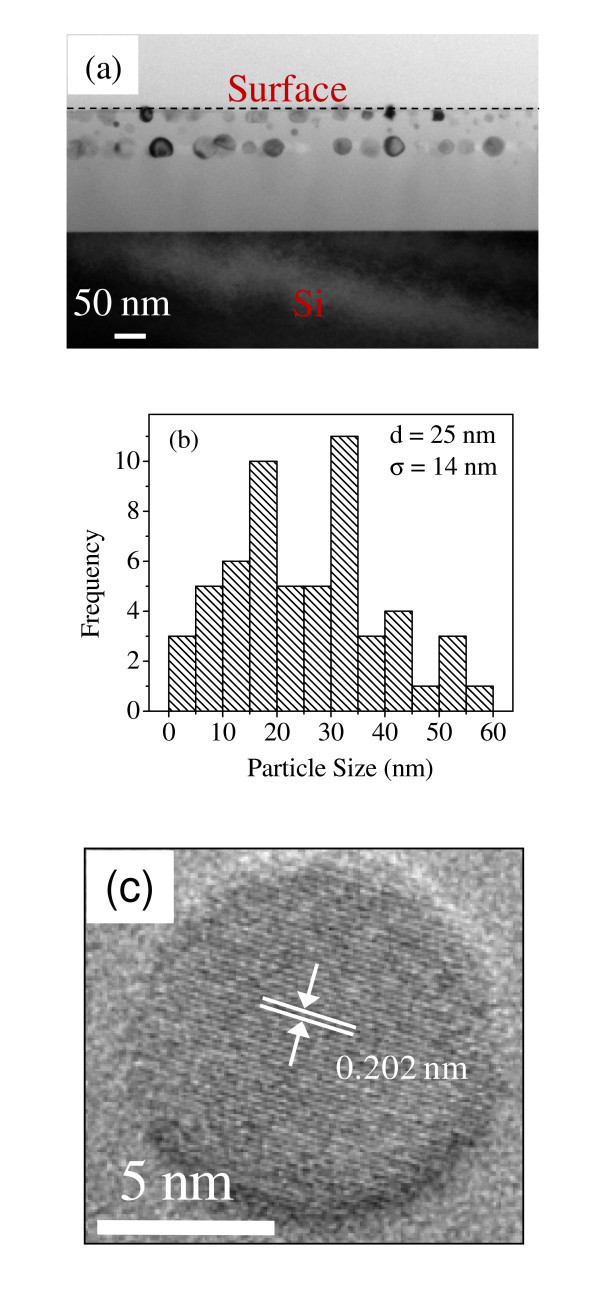
**Micro-structural study of pristine Ni-SiO**_**2 **_**film**. **(a) **Cross-sectional TEM micrograph of *pristine *Ni-SiO_2 _nanogranular film, **(b) **corresponding particle size histogram, and **(c) **high-resolution TEM micrograph of a spherical Ni nanoparticle

**Figure 2:**
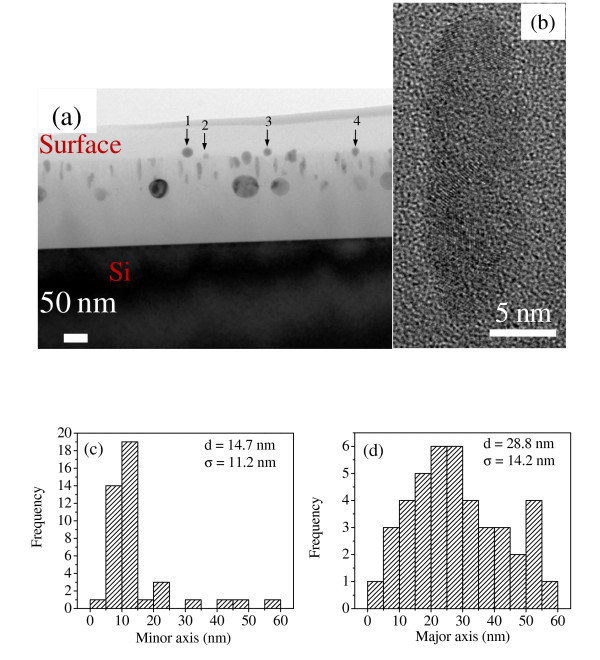
**Micro-structural study of irradiated Ni-SiO**_**2 **_**film**. **(a) **Cross-sectional TEM micrograph of *irradiated *Ni-SiO_2 _nanogranular film, **(b) **high-resolution TEM micrograph of an elongated Ni particle, and **(c)**, **(d) **histogram of minor and major axis lengths of elongated particles, respectively

Figure 1a shows the cross-sectional TEM micrograph of *pristine *Ni-SiO_2 _film and the corresponding histogram of particle sizes is shown in Figure 1b. It is clear from Figures 1a,b that the *pristine *film contains nearly spherical particles with a broad size distribution ranging from 3.8-60 nm with a mean particle size of ~25 nm. Figure 1c shows the high-resolution TEM micrograph of a particle evidencing its single crystalline nature and the measured lattice spacing of 0.202 nm corresponding to (111) plane of fcc Ni. Figure 2a shows the cross-sectional TEM micrograph of the *irradiated *film taking the direction of ion irradiation from top to bottom. It is clear from Figure 2a that most of the Ni NPs change from spherical to prolate shape with their major axis aligned along the direction of ion beam at a fluence of 5 × 10^13 ^ions/cm^2^. The elongated particles exhibit polycrystalline morphology, as apparent from high-resolution TEM micrograph (see Figure 2b). Figure 2c,d shows the histogram of major and minor axis length for prolate shape Ni particles. The mean major and minor axis lengths are 28.8 and 14.7 nm, respectively, estimated by considering all particles in Figure 2a. The mean aspect ratio for prolate-shaped particles is ~2. On comparing Figures 1a and 2a, it is observed that the smallest particles disappear after irradiation and shape deformation is completely suppressed for particles of size >14 nm. This confirms that the previous observations of shape deformation process is somewhat related to initial size of the nanoparticles, i.e., the bigger the particle the larger is its inertia against deformation/bigger particles require higher electronic stopping power for deformation [14-16]. Further, no deformation is observed for the free-standing Ni particles present at the surface of film (indicated by 1-3 in Figure 2a) and also those which are not surrounded by silica matrix completely (indicated by 4 in Figure 2a). This confirms previous observation by Pennikof *et al. *[38], which demonstrated the need of the surrounding matrix for shape deformation process upon comparison with free-standing particles. SHI irradiation is known for modification of materials due to removal of atoms from the surface of a material. This process is called electronic sputtering as it is governed by electronic stopping power at higher energies. Generally, a higher sputtering yield is observed for insulators (particularly silica) than metals [39-42], and this may be responsible for the removal of silica surrounding the surface Ni NPs in the *irradiated *film. TEM results indicate the dissolution of Ni particles much smaller than ion track in silica matrix (of which diameter will be discussed later), whereas the growth and elongation of relatively bigger particles by 120 MeV Au^+9 ^ions at a fluence of 5 × 10^13 ^ions/cm^2 ^and also a threshold size (14 nm) exists above which no shape deformation occurs under the studied beam parameters.

### Magnetic study

**Figure 3:**
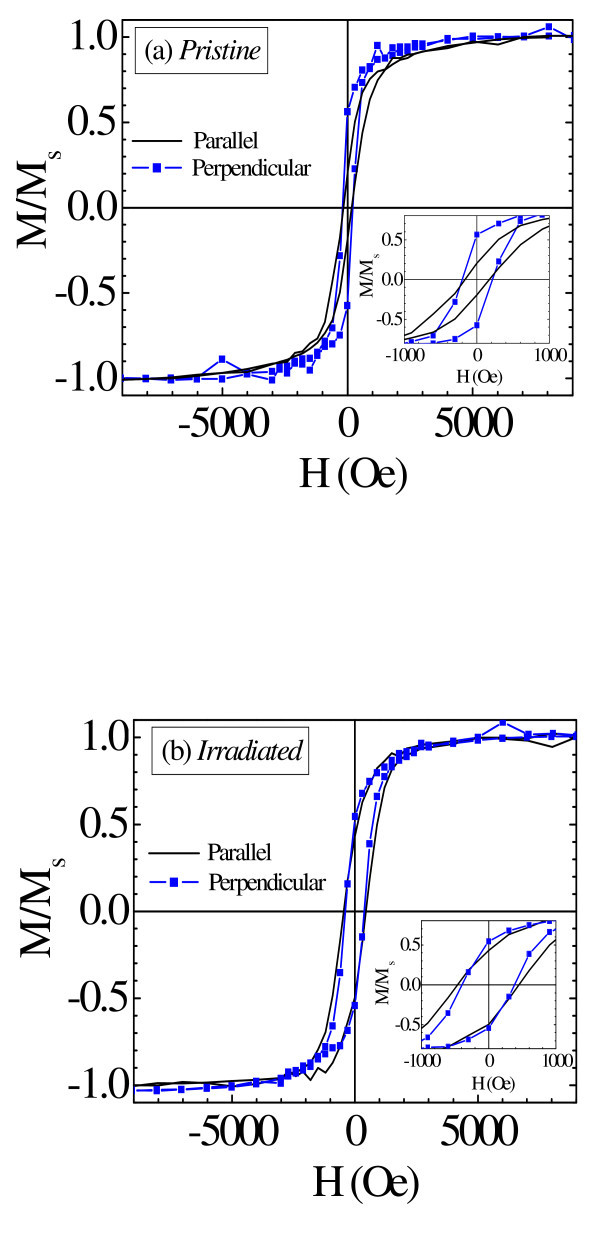
**M-H curve measured at 5 K**. For **(a) ***pristine *and **(b) ***irradiated *film with a maximum magnetic field of 20 kOe applied parallel and perpendicular to ion beam direction

In order to observe the effect of irradiation on magnetic properties, magnetization curves were measured at 5 K in a magnetic field applied both parallel (out-plane measurement) and perpendicular (in-plane measurement) to the ion beam direction. The M-H curves for *pristine *and *irradiated *film are shown in Figure 3a,b, respectively. The extracted coercivity (*H*_c_) and remanence ratio (*M*_r_/*M*_s_) from Figure 3a,b are given in Table 1. It is clear from Figure 3a that the *pristine *film has a small magnetic anisotropy with easy axis in the direction perpendicular to ion beam (in-plane). The origin of in-plane easy axis is the over-all thin film-like structure, i.e., anisotropy arising from the shape effect results in an in-plane easy axis, as similarly observed in case of Fe: SiO_2 _granular films [25,26]. The other factors like magneto-crystalline, magnetostriction and shape anisotropy may be neglected as *pristine *film is polycrystalline in nature and without stress as confirmed by X-ray diffraction studies (figure not shown) containing spherical Ni particles (see Figure 1a). However, after 120 MeV Au^+9 ^ion irradiation, the change in *H*_c _and *M*_r_/*M*_s _values is much larger in the direction parallel to Au ion beam than in the perpendicular direction, which can be correlated with the elongation/formation of prolate shape Ni particles in the beam direction. Hence, magnetic shape anisotropy appears in the elongated Ni NPs with easy axis in the direction of elongation. However, a macroscopic magnetic anisotropy with easy axis in the ion beam direction is not observed due to the existence of some spherical Ni particles in addition to deformed prolate particles in the *irradiated *film.

**Table 1:** Coercivity (*H*_c_) and remanence ratio (*M*_r_/*M*_s_) measured at 5 K for the *pristine *and *irradiated *Ni-SiO_2 _nanogranular film with magnetic field parallel and perpendicular to the 120 MeV Au^+^^9 ^ion beam direction.

Sample	Parallel		Perpendicular	
	***H***_**c **_**(Oe)**	***M***_**r**_**/*M***_**s**_	***H***_**c **_**(Oe)**	***M***_**r**_**/*M***_**s**_
*Pristine*	168	0.19	208	0.56
*Irradiated*	457	0. 45	388	0.54

### Simulations based on thermal spike model

In order to elucidate the anisotropic shape deformation of Ni NPs under SHI irradiation, we adopt the thermal spike model to simulate the temperature evolution around the Ni NPs. Here, we extend the thermal spike model to permit simulations for multiphase materials [14], considering the ion to pass through the center of Ni particle. In the thermal spike model [29-31], an incident heavy ion imparts its energy initially to target electrons and excites them to high temperature (within ~1-10 fs) and is subsequently transferred from hot electrons to lattice vibrations through electron-electron scattering (within ~100 fs) and then electron-phonon coupling, causing an increase in lattice temperature above the melting point of the target within 0.1-10 ps depending upon the target under consideration. After ~0.1-1 ns the thermal spike cools down to ambient conditions. This process can be described by a set of coupled thermal diffusion equations [43] for electronic and lattice subsystems.

(1) $$C_{ei}(T)\frac{\partial T_{e}}{\partial t}=\nabla [K_{ei}(T)\nabla T_{e}]+A(r,t)-g_{i}(T_{e}-T_{l}),$$

(2) $$\rho_{li}C_{li}(T)\frac{\partial T_{l}}{\partial t}=\nabla [K_{li}(T)\nabla T_{l}]+g_{i}(T_{e}-T_{l})$$

where *T*_e_, *T*_l_, *C*_e__*i *_(*T*), *C*_l__*i*_(*T*), *K*_e__*i*_(*T*) and *K*_l__*i*_(*T*) are the temperatures, the specific heats, and thermal conductivities of electronic (subscript *e*) and lattice (subscript *l*) subsystems, respectively; g_*i *_is the electron-phonon coupling constant; *ρ*_l__*i *_is the density of lattice, where *i *= Ni, SiO_2 _represents the Ni particle region and surrounding SiO_2 _region, respectively. *A*(*r*, *t*) is the energy density per unit time transferred from incident ions to the electronic subsystem at a distance *r *and at time *t *from ion path. As according to the thermal spike model the lattice temperature for times ~1-10 ps is more like the representation of the energy transferred to the lattice. Therefore, radial distribution of lattice temperature is simulated within 1, 5, and 10 ps of 120 MeV Au^+9 ^ion impact for Ni particles (2, 4, 6, 10, 15, 20, 30 nm) embedded in silica matrix. Table 2 shows the fitted values of the various parameters used for Ni [44] and silica [30,45] in the thermal spike model-based simulations.

**Table 2:** The fitted values of mass density (*ρ*), melting temperature (*T*_M_), vaporization temperature (*T*_V_), latent heat of fusion (*L*_M_), latent heat of vaporization (*L*_V_), lattice specific heat (*C*_l_), lattice thermal conductivity (*K*_l_) and electron-phonon coupling constant (*g*) for Ni and SiO_2 _used in the thermal spike simulations [30,44,45].

Parameter	Ni	**SiO**_**2**_
*ρ *(g cm^-3^)	8.9	2.62 (solid), 2.32 (liquid)
*T*_M _(K)	1,726	1,950
*T*_V _(K)	3,005	3,223
*L*_M _(J g^-1^)	290.3	142
*L*_V _(J g^-1^)	6,442	4,715
*C*_l _(J g^-1 ^K^-1^)	0.39 + 1.9 × 10^-4^T-3.3 × 10^-8^T^2^+3.8 × 10^-11^T^3^; (300 <*T *<*T*_m_),	0.65 + 3.297 × 10^-4^T; (300 <*T *<*T*_M_),
	0.62; (T > T_M_).	1.3-3 × 10^-7^*T*; (*T *> *T*_M_).
*K*_l _(WK^-1^cm^-1^)	3.4-1.3 × 10^-2^T+2.12 × 10^-5 ^T^2^- 1.5 × 10^-8^T^3^+3.6 × 10^-12^T^4^;	1 × 10^-3 ^(*T *> 300)
	(100 <*T *<*T*_M_), 0.5 (*T *> *T*_M_)	
*g*_l _(W cm^-3 ^K^-1^)	9.54 × 10^11^	1.25 × 10^13^

**Figure 4:**
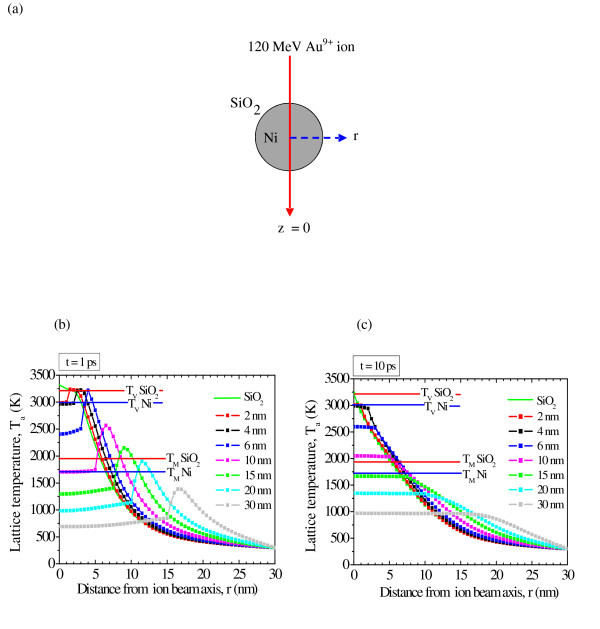
**Simulations based on thermal spike model**. **(a) **Schematic model for the thermal simulation of a Ni particle embedded in the SiO_2 _matrix irradiated by 120 MeV Au^+9 ^ion. **(b) **Calculated radial profile of lattice temperature in the z = 0 plane of bulk SiO_2 _and seven different spherical Ni nanoparticles (2, 4, 6, 10, 15, 20, 30 nm) embedded in silica after 1 ps and **(c) **10 ps of ion impact. The melting (*T*_M_) and vaporization (*T*_V_) temperature of SiO_2 _and Ni are also indicated

Figure 4a shows, schematically, a simplified two-dimensional model, in which a 120 MeV Au^+9 ^ion passes through the center of a spherical Ni particle embedded in silica matrix. Figure 4b,c shows the simulated radial distribution of the lattice temperature within 1 and 10 ps of 120 MeV Au^+9 ^ion impact, for bulk silica and Ni nanoparticles (diameter, 2-30 nm) embedded in a silica matrix. It is well studied that a latent track may result due to the rapid quenching of the molten lattice. Here in our case, the estimated molten region in silica is ~10 nm from simulation results and agrees well with the earlier published experimental results [30]. Thermal spike simulations cannot be applied to surface NPs which behave differently (temperature evolution and stress relaxation) from embedded NPs. The following observations are evident from Figure 4b, c: (1) For 0 < d ≤4 nm Ni particles temperature reaches up to its bulk vaporization temperature (*T*_v_) throughout its volume, so Ni atoms dissolve in the cylindrical molten silica track and promote the growth of neighboring bigger nanoparticles (>4 nm) by a ripening process, (2) For 4 < d ≤10 nm Ni particles, the lattice temperature of both Ni and surrounding silica rises above their respective melting points, so both Ni and silica are in molten state. Because of high electron-lattice coupling constant and low thermal conductivity of silica as compared to Ni, the temperature rise in silica is high as compared to metallic Ni even though incident ion energy is deposited to Ni and then coupled to silica, but thermal evolution itself does not give full explanation for deformation of metal NPs and hence shear stress also needs to be included as another factor in shape deformation. (3) For Ni particles having diameter >10 nm, Ni particle and surrounding silica both have temperature below their respective melting point and so retain their original shape. However, from TEM micrographs it is observed that Ni particles of size <14 nm are deformed and shape deformation is completely suppressed for particles of size >14 nm. The diameter in experiments does not match exactly with those obtained from thermal spike model-based simulations as pressure-dependent variation of thermodynamic parameters is neglected and size dependent variation of thermodynamic parameters is unknown, and hence the bulk values for Ni are used in the present case. The outcome of thermal spike simulations is that the lattice temperature of smaller size Ni particles increases much higher than lattice temperature of relatively bigger particles and experimental results are explainable within errors.

One may also think *ion hammering *as responsible mechanism for the elongation of Ni NPs. However, according to ion hammering mechanism, a large elongation is expected for smaller particles than relatively bigger particles, which contradicts our observation and hence rules out ion hammering effect. Klaumünzer *et al. *[46] also pointed out that hammering as an indirect mechanism alone is not sufficient to account for the observed deformation of solid NPs, i.e., the metallic NP must actively participate in the deformation process. In other words, elongation occurs only when the lattice temperatures of both metallic NP and the dielectric SiO_2 _exceed their respective individual melting temperatures, i.e., elongation of embedded NPs occur due to flow of metallic species into molten silica tracks [14,22]. According to the viscoelastic thermal spike model [27,28], the origin of anisotropic shape deformation of amorphous materials, e.g., silica is due to relaxation of shear stresses in the ion track region. These shear stresses are generated due to rapid thermal expansion of the ion-induced thermal spikes. The complete relaxation in the track region is assumed to take place when the ion track temperature exceeds a certain flow temperature (melting point). As for 4 < d ≤10 nm diameter Ni NPs this condition (melting of Ni as well as surrounding silica) is satisfied, so combination of stress effects in silica and thermal spike model gives a fairly good explanation for the elongation/shape deformation from spherical to prolate shape of Ni NPs.

## Conclusions

In conclusion, we report the elongation of Ni NPs parallel to each other embedded in silica matrix by 120 MeV Au^+9 ^ion irradiation at fluence of 5 × 10^13 ^ions/cm^2 ^with mean aspect ratio of ~2. Shape deformation is observed for particles <14 nm and is suppressed for particles >14 nm under studied beam parameters. Irradiation leads to formation of surface Ni particles without silica matrix and also not deformed, expected due to large electronic sputtering yield of silica. Large changes in coercivity (*H*_c_) and remanence ratio (*M*_r_/*M*_s_) are observed in the direction parallel to Au^+9 ^ion beam than in the perpendicular direction, which is due to the elongation/formation of prolate shape Ni particles in the beam direction. However, a macroscopic perpendicular magnetic anisotropy is not observed due to the existence of both spherical and deformed prolate shape Ni particles in the *irradiated *film. The experimental observations are well explained by thermal spike model-based simulations. Fabrication of *pristine *films with particles of average size in the range from 5 to 20 nm in order to control the macroscopic magnetic anisotropy with easy axis in the ion beam direction could be set as future perspective of this work.

## Competing interests

The authors declare that they have no competing interests.

## Authors' contributions

HK, SG, DKA and DK designed the experiments. HK and SG performed the experiments related to sample preparation and ion irradiation, AM performed the TEM analysis, SZ and HS performed the magnetic analysis, JPS performed the Thermal spike simulations. HK wrote the manuscript. All authors discussed the results and commented on the manuscript.
